# Advances in silica nanoparticles for agricultural applications and biosynthesis

**DOI:** 10.1007/s44307-025-00067-7

**Published:** 2025-04-28

**Authors:** Fei Li, Yuxi Hou, Lu Chen, Yimin Qiu

**Affiliations:** 1 National Biopesticide Engineering Research Centre, Biopesticide Branch of Hubei Innovation Centre of Agricultural Science and Technology, Hubei Biopesticide Engineering Research Centre, Hubei Academy of Agricultural Sciences, Wuhan, Hubei 430064 China; 2 College of Life Sciences, Hubei University, Wuhan, Hubei 430062 China

**Keywords:** Nanotechnology, SiO_2_ NPs, Agricultural applications, Biosynthesis, Stress resistance

## Abstract

Nanotechnology has emerged as a revolutionary force in modern agriculture, opening new avenues for crop enhancement and sustainable farming practices. This review systematically evaluates the roles of silica nanoparticles (SiO_2_ NPs) in agricultural applications, with particular emphasis on their biosynthesis pathways and functional mechanisms. SiO_2_ NPs have demonstrated considerable potential to enhance crop resilience against both biotic (pathogens, pests) and abiotic (heavy metals, salinity, drought) stresses through phytohormonal regulation, defense gene activation, and metabolic modulation. As nanocarriers, these particles enhance pesticide and fertilizer delivery accuracy, reduce environmental contamination, and promote plant growth. Biosynthesis methods of SiO_2_ NPs range from conventional physical–chemical techniques to eco-friendly green approaches, including the utilization of biological cells/extracts, natural biomaterials, and peptide templates. Although green synthesis offers sustainability advantages, the agricultural adoption of SiO_2_ NPs faces critical challenges, such as insufficient understanding of their long-term environmental persistence and ecotoxicological impacts, high production costs related to green synthesis, and incomplete regulatory frameworks. Addressing these challenges is essential to enable their broader use in agriculture.

## Introduction

Global agricultural development faces unprecedented challenges due to escalating food demand driven by population growth and climate change-induced adversities. These challenges manifest in three critical areas: (1) biotic stress, where pathogens (bacteria, fungi, viruses) and pests threaten crop health, leading to 10%–40% annual yield losses in major crops (e.g., wheat, rice, maize) ‌and quality degradation (Fu et al. 2020; Savary et al. 2019); (2) abiotic stresses, with droughts, salinity, and extreme temperatures reducing crop productivity by over 50% under conventional farming practices (Rakkammal et al. 2022); and (3) environmental degradation‌, exemplified by soil acidification (e.g., pH of the surface soil in the Chengdu Plain of China decreased by 1.2 units during 1980 s–2010 s) and water eutrophication caused by excessive agrochemical use (Adisa et al. 2019; Li et al. 2020).

Nanotechnology has emerged as a revolutionary paradigm to address these challenges. Silicon (Si), constituting ~ 26% of Earth's crust, plays an essential role in plant physiology as a beneficial element (Guntzer et al. 2012). Among its various forms, silica nanoparticles (SiO_2_ NPs) are nanoscale materials with diameters between 1–100 nm. Reduced particle size sharply increases their specific surface area, triggering a surface-interface effect (Rastogi et al*.*, 2019). This enhances surface-active sites and chemical reactivity (Yan et al. 2024). Moreover, smaller particle sizes enable SiO_2_ NPs to penetrate plant cell walls and organelles, facilitating direct interaction with cellular components (Pan et al*.*, 2023). These properties establish SiO_2_ NPs as versatile platforms for agricultural innovation.

Recent studies ‌demonstrate‌ the remarkable potential of SiO_2_ NPs as precision-engineered solutions in agriculture. They ‌exhibit exceptional efficacy in combating biotic and abiotic stresses in plants, achieved through induction of defense responses, suppression of phytopathogens, and enhancement of stress tolerance via various physiological mechanisms (Cáceres et al. 2019; Verma et al. 2022). As intelligent carriers‌, these nanomaterials revolutionize pesticide/fertilizer delivery by improving payload utilization efficiency and minimizing environmental contamination (Kong et al*.*, 2021, Mathur & Roy, 2020). Advances in green synthesis protocols further strengthen‌ their sustainability profile, offering eco-friendly alternatives to conventional chemical synthesis (Bazzi et al. 2023).

Despite their broad potential, the agricultural application of SiO_2_ NPs remains constrained by several critical challenges spanning environmental, economic, and regulatory domains. Environmentally, uncertainties regarding degradation kinetics, bioaccumulation patterns, and interactions with co-pollutants pose risks to non-target organisms and overall ecosystems stability (Yadav et al. 2022). Economically, the high production costs, driven by energy-intensive conventional synthesis methods and resource-demanding biosynthetic routes, along with low conversion efficiency and inconsistent batch quality, impede commercial scalability. Regulatory heterogeneity further complicates global deployment, with divergent definitions, risk assessment frameworks, and certification requirements across regions (Kumari et al. 2023).

This review systematically evaluates three critical dimensions of SiO_2_ NPs in agriculture: (1) their applications in crop protection, nutrient management, and stress alleviation; (2) the molecular mechanisms underlying their bioactivity; and (3) innovations in sustainable production technologies. The analysis ‌provides actionable insights for optimizing SiO_2_ NP-based interventions to advance precision agriculture and sustainable cropping systems. Furthermore, the review critically examines implementation challenges, including environmental risk quantification, cost-efficient scale-up strategies, and international regulatory harmonization, and proposes suggestions for future research priorities in sustainable nanopesticide development.

## Applications of SiO_2_ NPs in agriculture

**Fig. 1:**
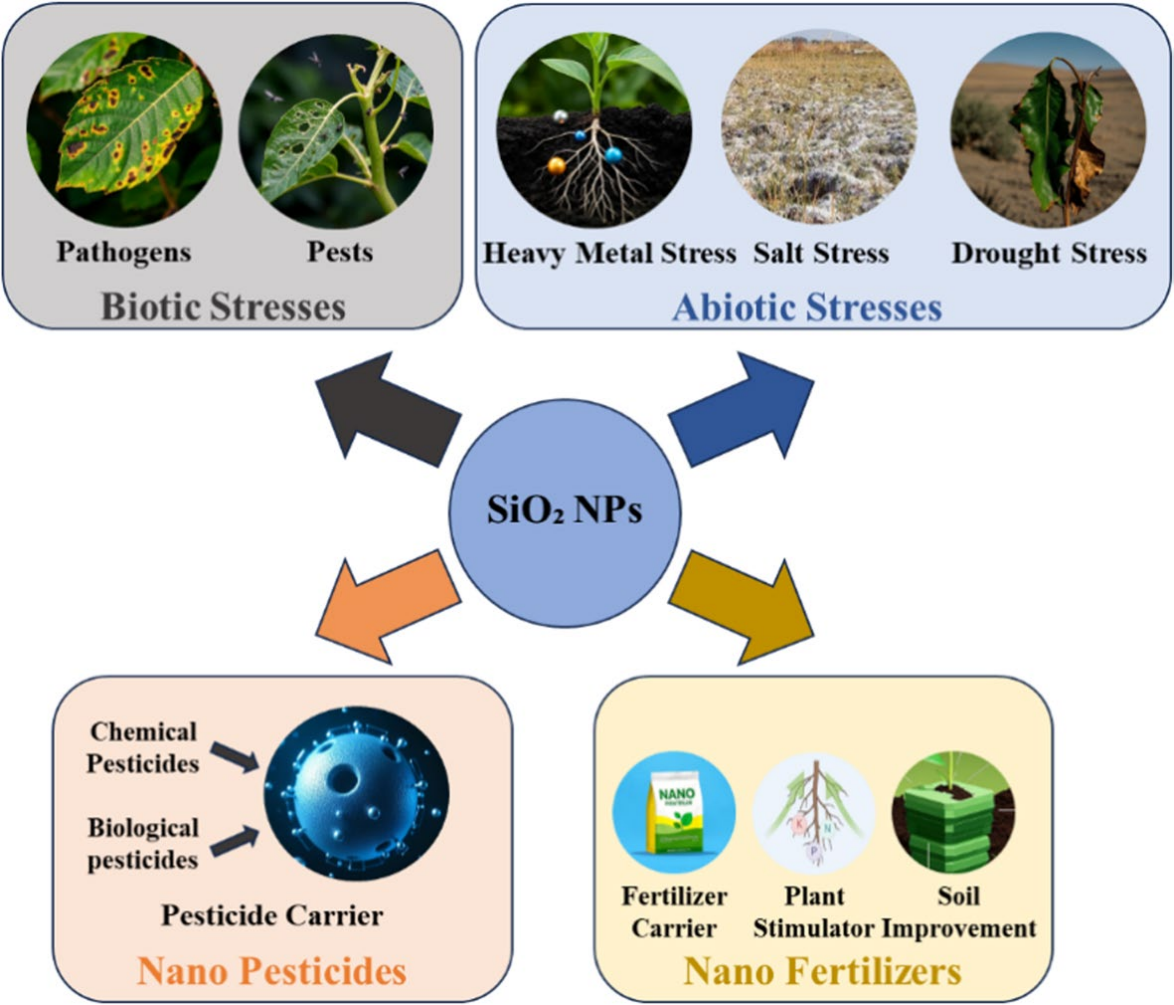
Application of SiO_2_ NPs in agriculture

Modern agricultural systems face dual challenges of intensifying environmental stressors and inefficient agrochemical utilization, both of which directly impair crop growth, reduce yields, and exacerbate pollution. SiO_2_ NPs have emerged as multifunctional solutions to these issues, enhancing crop resilience while optimizing resource-use efficiency, thereby advancing‌ sustainable agriculture (Fig. 1).

### Role in coping with crop biotic stresses

#### SiO_2_ NPs conferring resistance to plant pathogens

Upon application, SiO_2_ NPs penetrate root cell walls, diffuse through plasmodesmata, and are transported to aerial plant tissues, such as stems and leaves, via the xylem. These nanoparticles exist in various aggregation states (e.g., phytoliths and amorphous silica deposits) and function as biostimulants, triggering systemic defense responses against pathogens (Mathur & Roy, 2020). Evidence has shown their efficacy in diverse crops, including rice, wheat, carrot, mango, sorghum, and tomato, with reported reductions in disease index ranging from 60 to 87% (Debona et al. 2017, JUNIOR et al*.*, 2019, Ahamad & Siddiqui 2021). For instance, SiO_2_ NPs suppress major rice diseases, such as sheath blight, blast, brown spot, leaf blight, stem rot, and grain discoloration (Gabr et al*.*, 2017, Du et al. 2022). They control root rot, leaf spot, rust, and powdery mildew in vegetables like cucumbers and peas, as well as in sugarcane and wheat (Souri et al. 2021). Additionally, SiO_2_ NPs mitigate early blight in tomato caused by *Alternaria solani*, bacterial necrosis in mango roots caused by *Pseudomonas syringae*, and anthracnose in sorghum caused by *Colletotrichum sublineolum* (Resende et al. 2013, Derbalah et al. 2018, Gutiérrez‐Barranquero et al*.*, 2012).

The mechanisms by which SiO_2_ NPs confer resistance to plant pathogens involve both physical and biochemical processes. Physically, SiO_2_ NPs accumulate in epidermal tissues and form silica-bilayer complexes by cross-linking with hemicellulose in cell walls, blocking pathogen invasion (Luyckx et al. 2017). Biochemically, SiO_2_ NPs bind to receptors on plant cell surfaces, initiating signal transduction cascades that activate host defense mechanisms. These responses include the upregulation of defense-related enzymes (e.g., 1,3-beta-glucanase, peroxidase, chitinase, glutathione reductase, lipoxygenase, polyphenol oxidase, and phenylalanine lyase) and synthesis of antimicrobial compounds like phenols, flavonoids, diterpenoids, and other plant antibiotics. Simultaneously, SiO_2_ NPs modulate key hormone signaling pathways, including salicylic acid (SA), jasmonic acid (JA), and ethylene (ET) pathways (Wang et al. 2017, Kandhol et al., 2022). Such integrated defense responses were evidenced in tomato trials against *Ralstonia solanacearum*, where SiO_2_ NPs treatment resulted in a 1.79–2.58-fold increase in peroxidase (POD) activity, a 1.46–1.52-fold elevation in superoxide dismutase (SOD), and 1.59–1.64-fold increase in catalase (CAT) enzyme activities, along with upregulation of *PR- 1*, *PR- 5*, and PAL genes associated with SA signaling in tomato leaves (Wang et al. 2022).

#### SiO_2_ NPs conferring resistance to pests

SiO_2_ NPs exhibit broad-spectrum insecticidal activity and are increasingly employed‌ to combat pest pressures in agriculture. When applied to the cuticles of insects or their larvae, the nanoparticles adsorb onto lipid layers, interfering with spiracular gas exchange and tracheal contraction rhythms, leading to respiratory disorders. Alternatively, the nanoparticles may abrade the epicuticular wax layer, causing dehydration-induced mortality (Barik et al. 2008). Following ingestion, they may physically damage the digestive tract or generate free radicals via siloxane bond cleavage, disrupting insect metabolism and exerting an indirect insecticidal effect (Ayoub et al. 2017). Notably, their physical mode of action circumvents conventional pesticide resistance mechanisms.

Rouhani et al. (2012) reported‌ that SiO_2_ NPs achieved *LC*_50_ values of 1.03 g/kg (larvae) and 0.68 g/kg (adults) against the cowpea seed beetle (*Callosobruchus maculatus*). Field trials by‌ Thabet et al. (2021) demonstrated 100% population reduction for cotton leafworm (*Spodoptera littoralis*), aphid (*Aphis craccivora*) and *Liriomyza trifolii* on faba beans using 75–425 mg/L SiO_2_ NPs within 7–15 days, with aphids showing the highest susceptibility‌. Additionally‌, Elsadany et al. (2015) ‌observed‌ a sigmoidal dose–response in SiO_2_ NPs'acaricidal activity against the spider mite (*Tetranychus cucurbitacearum*), achieving 78.91% mortality at 450 ppm.

The insecticidal efficacy of SiO_2_ NPs is critically influenced by‌ their physicochemical properties. Size-dependent toxicity was evident in the rice weevil (*Sitophilus oryzae*), where 15–30 nm particles induced 97% mortality versus 23% for 100–400 nm particles at 2 g/kg (Debnath et al*.*, 2011). Morphological engineering via surfactant templates significantly enhanced their bioactivity. SiO_2_ NPs synthesized using Triton X- 100, cetyltrimethylammonium bromide (CTAB), and polyvinylpyrrolidone (PVP) exhibited 96.6%, 93.3%, and 90.0% mortality in *Spodoptera littoralis*, ‌respectively, while commercial SiO_2_ material showed no entomotoxic effects within the first 3 days (Ayoub et al. 2017). Surface functionalization with‌ 3-mercaptopropyltrimethoxysilane (MPTMS) ‌and‌ hexamethyldisilazane (HMDS) ‌increased‌ larval mortality in cabbage armyworm (*Spodoptera litura*) to 64% and 58% within 24 h, ‌likely through enhanced lipid adsorption (Debnath et al. 2012).

### Role in coping with crop abiotic stresses

#### Heavy metal stress

Heavy metal stress exerts detrimental effects on plants by suppressing growth, disrupting photosynthesis, and inducing oxidative damage via the overproduction of reactive oxygen species (ROS) (Ghori et al. 2019). SiO_2_ NPs mitigate‌ these impacts ‌through multi-level defense modulation‌. At the photosynthetic level, they enhance‌ chlorophyll and carotenoid accumulation, improving‌ photosynthetic capacity under metal stress (Okeke et al. 2023). At the antioxidant defense level, SiO_2_ NPs boost antioxidant enzyme activities (e.g., SOD, POD, CAT, ascorbate peroxidase (APX)), and promote the accumulation of antioxidant substances like glutathione (GSH). These effects enable plants to scavenge excessive ROS, mitigate heavy metal-induced oxidative stress (Roy et al. 2025). Additionally, they regulate genes involved in heavy metal transport, such as inhibiting *OsHMA2* and *OsHMA3* in rice, as well as *ST1* and *MT* in *Brassica napus*, thereby reducing metal uptake and accumulation (Kim et al. 2014; Huang et al. 2024).

These mechanisms synergistically enhance‌ plant resilience. For instance, Ahmed et al. (2023) demonstrated that SiO_2_ NPs alleviated cadmium (Cd) stress in rapeseed (*Brassica napus* L.) by increasing photosynthetic efficiency. SiO_2_ NPs-treated rapeseed exhibited 31.8% higher chlorophyll content and 38% improved net photosynthetic rate under Cd stress. Meanwhile, SiO_2_ NPs reduced oxidative stress through the activation of antioxidant enzymes (19.1% increase in SOD, 33.4% in POD, 14.4% in CAT, and 33.8% in APX), thereby lowering ROS generation. Moreover, SiO_2_ NPs treatment decreased Cd translocation by 25.4% in roots and 33.3% in shoots.

Similarly, Saadony et al. (2021) reported that 5.0 mM SiO_2_ NPs induced dose-dependent increases in chlorophyll (by 12.3%) and carotenoid (by 16.5%) levels in common bean plants (*Phaseolus vulgaris*) grown on Cd–Pb-Ni contaminated soil. The nanoparticles induced 40.9%–178% increase in enzymatic antioxidants (SOD, POD, CAT, APX) and non-enzymatic antioxidants (proline), reducing bioavailable metals by 31%–58%, while increasing dry weight by 22.5% and shoot length by 21.8%.

#### Salt stress

Salt stress induces multifaceted plant homeostasis disruption ‌via‌ osmotic imbalance and ion toxicity, compromising photosynthesis, enzymatic catalysis, and hormonal regulation. ROS overproduction further exacerbates oxidative damage to cellular macromolecules, including lipids, proteins, and DNA, thereby hindering plant growth and development, which may ultimately reduce crop yields (Ondrasek et al. 2022).

SiO_2_ NPs mitigate‌ salt stress ‌through dual protective mechanisms‌. Morphologically, SiO_2_ NPs ‌enhance‌ cell wall thickness ‌and‌ mechanical strength ‌by‌ epidermal deposition, effectively counteracting osmotic pressure and water loss (Dhiman et al. 2021). This structural modification was evidenced by Avestan et al. (2019), who demonstrated that SiO_2_ NPs-treated strawberry plants exhibited enhanced epicuticular wax accumulation, reducing water loss under NaCl stress. Metabolically‌, SiO_2_ NPs stimulate the biosynthesis of osmolytes, such as proline, soluble sugars, and glycine betaine, maintaining intracellular turgor pressure and osmotic balance (Liang et al. 2023). Specifically‌, lentil seedlings treated with SiO_2_ NPs exhibited significant upregulation of sugar and osmolytes under NaCl stress, correlating with improved water retention capacity and enhanced germination ‌and‌ biomass (Sarkar et al. 2022).

Ion homeostasis regulation by SiO_2_ NPs involves‌ Na^+^ influx suppression ‌and‌ K^+^ selectivity enhancement ‌via‌ membrane transporter modulation (Yin et al. 2016). In salt-stressed cucumbers‌, SiO_2_ NPs treatment resulted in a 7.2%–34.5% decrease in cytosolic Na^+^ concentration and a 14%–47.4% increase in K^+^/Na^+^ ratio (Alsaeedi et al. 2018). This ion selectivity directly impacts membrane potential stabilization, enzyme activity optimization, and overall metabolic functionality. Similarly‌, Singh et al. (2022) documented that SiO_2_ NPs alleviated NaCl-induced electrolyte leakage in wheat leaves and roots by up to 26% and 43%, indicating restoration of membrane integrity.

#### Drought stress

Drought stress ‌induces physiological and biochemical dysregulation in plants by disrupting water balance and metabolic dysfunction. Physiological constraints primarily arise from impaired water uptake and translocation, which lead to stomatal closure to minimize water loss through transpiration. This, in turn, suppresses photosynthetic activity and nutrient assimilation (Seleiman et al. 2021). Concurrently, ROS overproduction overwhelms cellular antioxidant defenses, ‌causing‌ oxidative damage to membranes and biomolecules.

SiO_2_ NPs demonstrate‌ broad-spectrum drought mitigation across diverse plant species, including cereals (rice, wheat, maize), horticultural plants (tomato), and legumes (broad bean). Their protective mechanisms involve coordinated regulation of water relations and stress-responsive pathways. In experiments, SiO_2_ NPs enhanced hydraulic conductivity by stimulating root architectural modifications, such as increasing the root length to 2 times, thereby expanding the absorptive surface area (Du et al. 2022). Stomatal aperture modulation further reduced transpiration rates, optimizing water retention capacity (Malik et al. 2021). Additionally, they stabilized thylakoid membranes and elevated chlorophyll biosynthesis: chlorophyll a content was increased by 9.1%–16.7%, and chlorophyll b content was increased by 100%–166.7% under treatment with 900 mg/L SiO_2_ NPs, ensuring sustained photochemical efficiency under drought (Raza et al. 2023). Biochemically, SiO_2_ NPs augment osmoregulation by accelerating the accumulation of compatible solutes, particularly proline (increased by 25.7%–133.8%), which collectively lower osmotic potential and maintain turgor pressure (Abdo et al*.*, 2024). Simultaneously, they reinforce the activities of SOD and CAT, and promote the accumulation of non-enzymatic scavengers (e.g., ascorbate, glutathione), effectively neutralizing ROS overproduction and mitigating oxidative injury (Esmaili et al. 2022).

These adaptations demonstrate synergistic enhancement of drought resilience. ‌In broad beans, exposure to 1.5 mM SiO_2_ NP ‌increased‌ net photosynthetic rate by 18.1%, elevated relative water content by 7.3%, ‌and boosted nutrient uptake by 22.6%–120%, ‌while reducing‌ hydrogen peroxide (H_2_O_2_) and superoxide radical (O_2_^−^) levels by 39% and 16.9% respectively ‌through‌ redox homeostasis regulation (Desoky et al*.*, 2021).

### Applications in the fields of pesticides and fertilizers

#### SiO_2_ NPs as multifunctional pesticide delivery platforms

SiO_2_ NPs possess unique physicochemical properties. In particular, mesoporous variants can function as advanced delivery systems for a wide range of agrochemical formulations. Their high surface-area-to-volume ratio, tunable pore architecture, and modifiable surface chemistry facilitate‌ bioactive compounds encapsulation via physisorption, covalent conjugation, or matrix entrapment strategies (Kong et al*.*, 2021). This nano-engineering approach significantly enhances payload stability against environmental degradation while providing spatiotemporal control over active ingredient release kinetics. Notably, SiO_2_ NPs exhibit‌ broad compatibility with chemical pesticides and biopesticides, offering a unified platform for precision agrochemical delivery.

The prolonged and unsustainable application of conventional chemical pesticides has led to escalating ecotoxicological impacts and environmental contamination. The nano-biointerface characteristics of SiO_2_ NPs present a transformative solution by facilitating targeted pesticide deposition and translocation within plant tissues, thereby improving their utilization efficiency and reducing residual pesticide runoff. Their nanoscale dimensions (< 200 nm) promote cuticular permeation and stomatal entry, as evidenced by size-dependent translocation efficiency of pyraclostrobin, where 15 nm particles showed 2.5-fold higher pyraclostrobin mobility than 200 nm counterparts in cucumber roots (Xu et al. 2021). Foliar formulations‌ ‌with‌ SiO_2_ NPs achieved a‌ 89.2% ‌higher‌ rainfastness ‌on‌ cucumber leaves (Zhao et al. 2018). Integration of smart gatekeeper systems with SiO_2_ NPs enables precise pesticide delivery, further improving pesticide utilization efficiency. Pectin-coated mesoporous SiO_2_ NPs selectively released prochloraz in response to *Magnaporthe oryzae*-secreted pectinases, demonstrating prolonged antifungal efficacy (> 14 days) and improved uptake and translocation performance in rice tissues (e.g., the concentration of prochloraz in rice leaves increased by 1.5–7 times after treatment for 3–14 days) (Abdelrahman et al. 2021). These attributes synergistically improve pesticide utilization efficiency while reducing environmental leakage, aligning with sustainable agricultural goals.

Biopesticides (e.g., rotenone, avermectin derivatives and dsRNA) offer environmental advantages but exhibit inherent instability under ambient conditions. For instance, the light-sensitive emamectin benzoate (EB) showed improved photostability (2.25-fold increase) when encapsulated in amino-functionalized rough mesoporous silica nanoparticles coated with poly maleic anhydride (PMA). Notably, this formulation retained 37% insecticidal activity after 21 days, approximately fourfold higher than that of pristine EB (Yu et al. 2024). Amino-functionalized SiO_2_ NPs complexed with dsRNA targeting Potato virus Y (PVY) coat protein-maintained RNA integrity for 14 days while facilitating systemic silencing of viral replication machinery (Xu et al. 2023b).

Accumulating evidence demonstrates that SiO_2_ NPs-mediated delivery systems effectively mitigate the intrinsic limitations of both chemical and biopesticides, while exhibiting superior efficacy and sustainability over conventional formulations. The convergence of stimuli-responsive release, enhanced bioavailability, and environmental resilience establishes SiO_2_ NPs as transformative tools for next-generation crop protection strategies.

#### Applications in the field of fertilizers

Conventional fertilizer application faces critical inefficiencies, with over 50% of nutrient loss occurring through physicochemical dissipation pathways such as runoff, leaching, and degradation (Liu et al. 2024). This suboptimal nutrient delivery necessitates excessive chemical inputs, exacerbating environmental eutrophication and reducing crop productivity (Zulfiqar et al. 2019). As a transformative nanofertilizer, SiO_2_ NPs reconfigure nutrient delivery through three synergistic mechanisms: enhanced fertilizer utilization, physiological processes stimulation, and rhizosphere microecology modulation.

SiO_2_ NPs serve as effective carriers by encapsulating macronutrients (N, P, K) within their porous matrices, decoupling nutrient availability from environmental conditions. This encapsulation improves fertilizer solubility and enables‌ controlled release, ‌reducing‌ application frequency ‌and‌ nutrient loss (Goswami et al. 2022). For example‌, urea-loaded mesoporous SiO_2_ NPs extended nitrogen release duration by 5 folds compared to conventional formulations (Wanyika et al. 2012). Similarly, N-P-K co-loaded nanocomposites ‌achieved‌ sustained nutrient release over 30 days, whereas‌ conventional fertilizers depleted‌ nutrients within 7 days (Rahman et al*.*, 2024).

Beyond carrier functionality, SiO_2_ NPs directly enhance‌ plant physiology (Awad-Allah 2023). In cucumber plants, SiO_2_ NPs application led to increases in plant height (by 29.4%), leaf number (by 50%), and fruit yield (134.8%). They increased cell wall strength and elasticity during growth extension, slowed down Na^+^ uptake (by 7.65%), increased the K^+^ uptake (by 111.08%) (Yassen et al*.*, 2017). Furthermore, Roohizadeh et al. (2015) found that 1.5 mM SiO_2_ NPs increased broad bean germination rate by about 12.5% and hypocotyl length by about 9%, likely through‌ improved plant water status and photosynthetic efficiency.

At the soil-microbe interface, SiO_2_ NPs act‌ as edaphic conditioners by modifying soil physicochemical parameters. Field studies on maize demonstrated SiO_2_ NPs significantly enhanced the microbial population in rhizosphere soil, particularly enriching nitrogen-fixing bacteria and phosphate-solubilizing bacteria by around 53.3% and 52%, respectively, as well as increasing soil silica content by 140% (Rangaraj et al. 2014). Foliar application of SiO_2_ NPs elevated *Paenibacillus* abundance by 16% at a concentration of 5 mg per plant in pakchoi soil (Tian et al*.*, 2020). Such modifications synergize‌ with microbial diversity ‌to establish‌ nutrient cycling feedback loops, thereby ‌enhancing‌ crop biomass (Sharma & Kumar 2024; Rajput et al. 2021).

## Biosynthesis methods of SiO_2_ NPs

**Fig. 2:**
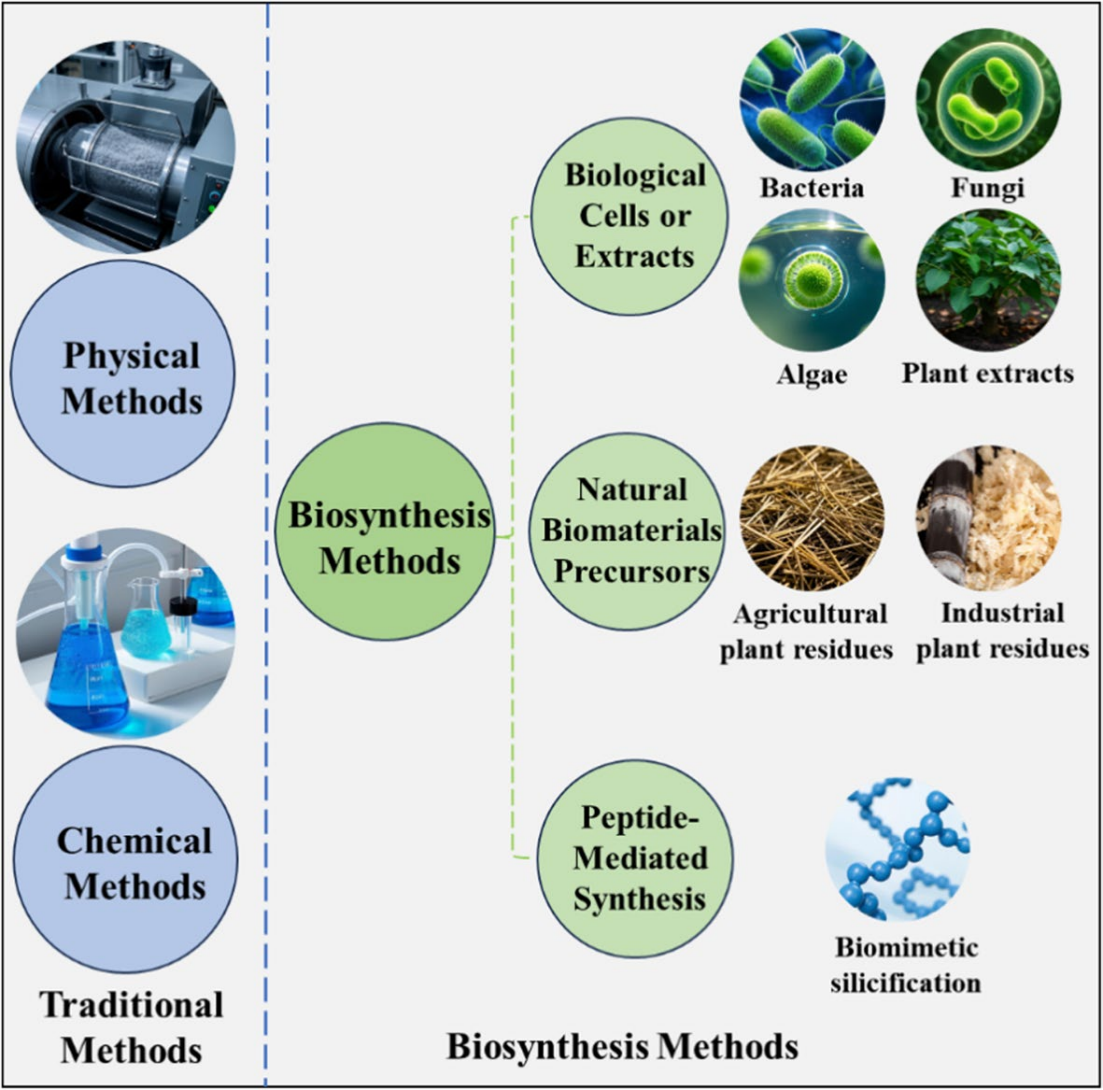
Traditional and biosynthesis methods of SiO_2_ NPs

The synthesis of SiO_2_ NPs is widely explored using traditional physical and chemical methods. However, these conventional approaches ‌require‌ high energy consumption and generate‌ chemical pollutants, which pose challenges to sustainability and environmental protection. ‌Recent advances in biosynthesis methods utilize biological cells or extracts, natural biological materials as precursors, and peptide-mediated biomimetic silicification ‌to achieve‌ resource-efficient ‌and‌ eco-friendly synthesis, ‌thereby addressing‌ the limitations of conventional techniques (Fig. 2).

### Overview of traditional synthesis methods

#### Physical methods

Mechanical comminution remains‌ a conventional physical synthesis method for SiO_2_ NPs, utilizing high-energy collisions from ball mills or ultrasonic devices to break bulk silica into nanoscale particles (Stopic et al. 2021). This approach is operationally simple and scalable for industrial applications without complex chemical steps. However, it struggles with precision control, often yielding particles with broad size distributions and irregular morphologies due to uncontrolled fracture dynamics. Additionally, prolonged mechanical processing introduces impurities (such as metallic residues) and causes structural defects, ‌reducing‌ colloidal stability and functional performance. These inherent limitations underscore the need for supplementary techniques to enhance purity and uniformity in mechanically synthesized SiO_2_ NPs.

#### Chemical methods

Chemical synthesis routes for SiO_2_ NPs rely‌ on controlled molecular interactions to engineer‌ tailored nanostructures. Techniques such as microemulsion, chemical vapor deposition (CVD), sol–gel, and precipitation offer varying balances of precision and scalability.

The ‌microemulsion method‌ confines silicon precursor reactions within surfactant-stabilized aqueous nanodroplets (5–50 nm diameter), allowing for precise size control under mild conditions. Nevertheless‌, it ‌demands‌ excessive organic solvents (25–100 L/kg) and multi-step purification, raising environmental and scalability concerns (Finnie et al. 2007).

CVD method uses gas-phase precursors (e.g., SiH_4_, SiCl_4_) that decompose or react on heated substrates (800–2000 °C), producing‌ high-purity SiO_2_ films or nanoparticles with sub-nm thickness uniformity. Despite achieving exceptional crystallinity and monodispersity, CVD requires specialized vacuum systems and energy-intensive thermal cycles, resulting in prohibitive costs for industrial-scale adoption (Silva 2004).

In the ‌sol-gel process‌, silicate esters (such as tetraethyl orthosilicate) undergo hydrolysis and polycondensation in solution to form a porous gel network, which then calcine into high-surface-area SiO_2_ NPs (100–1000 m^2^/g). This technique features mild reaction conditions, ease of operation, and the capacity to prepare high-purity SiO_2_ NPs with large surface areas. However, extended reaction durations (1–7 days) and the risk of irreversible agglomeration during drying stages frequently compromise structural homogeneity, affecting the quality of the final product (Trewyn et al., 2007).

The ‌precipitation method‌ involves the acid-induced gelation of Na_2_SiO_3_ solution, followed by filtration and calcination, yielding SiO_2_ NPs at a relatively low cost. However‌, rapid nucleation in this system often leads to polydisperse particle distributions and the presence of residual ionic impurities, requiring additional purification steps for advanced applications (Jal et al. 2004).

### Detailed description of biosynthesis methods

#### Synthesis using biological cells or extracts

Use of biological cells or extracts for SiO_2_ NP synthesis emerges‌ as an innovative green approach, leveraging the inherent molecular complexity and enzymatic activities of biological systems. Diverse bioresources, including bacteria, fungi, algae, and plant-derived phytochemicals (such as proteins, polyphenols, terpenoids, and polysaccharides), serve as multifunctional bio-templates or provide redox-active molecules that facilitate nanoparticle formation (Mohanpuria et al. 2008). At the molecular level, specific reductases present in these biological matrices convert‌ silicate precursors (e.g., sodium silicate) into amorphous silica nuclei. Surface-exposed functional groups (-OH, -COOH, -NH_2_) from biomolecules coordinate with silica nuclei, acting as capping agents around the nanoparticles. These biomolecular functional groups not only stabilize the nanoparticles but also enhance their biocompatibility and overall functionality (Shah et al. 2015).

This bio-mediated process operates under mild conditions, typically at ambient temperature (20–30 °C) and atmospheric pressure, which minimizes energy consumption and eliminates toxic byproducts generation. The resultant SiO_2_ NPs exhibit intrinsic bioactivity, making them ideal ‌for agricultural applications. For instance, yeast-mediated synthesis ‌produced‌ spherical amorphous nanoparticles (40–70 nm) ‌at‌ 29 °C, ‌achieving‌ near-zero environmental contamination (Zamani et al. 2020). *Bryophyllum pinnatum* leaf extract-capped nanoparticles enhanced shoot length and root length of *Vigna radiata* by‌ 7.5% and 50% respectively ‌at‌ 5 μg/mL, demonstrating‌ their nano-fertilizer potential (Sankareswaran et al. 2022). Systematic cytotoxicity assessments demonstrated the biosafety of SiO_2_ NPs produced using *Pseudomonas fluorescens*, *Trichoderma atroviride*, and *Streptomyces griseus*. These assessments revealed that zebrafish embryo survival rates were 94% ‌at 125 µg/mL ‌for‌ bio-synthesized nanoparticles, ‌outperforming‌ chemically synthesized SiO_2_ NPs (26.67% survival at‌ 200 µg/mL) (Natesan et al*.*, 2021, Duan et al. 2013).

#### Synthesis using natural biomaterials as precursors

Silicon-rich biomass-derived synthesis represents a sustainable pathway for SiO_2_ NPs production. Agricultural and industrial residues such as monocotyledonous crop byproducts (e.g., sorghum, rice husks, corn stalks) and processing wastes (e.g., bamboo leaf ash, sugarcane bagasse), contain silicon contents exceeding 50% by mass, with some species holding silica over 90% (Setiawan & Chiang 2021). Through sequential drying (100–500 °C), alkaline delignification (using 2–5 M NaOH), and mechanical milling (particle sizes of raw materials < 100 μm), these low-cost biological resources are converted into high-purity silica precursors while simultaneously achieving waste valorization.

This biosynthesis approach offers three advantages over conventional chemical methods. Firstly, it utilizes abundant raw materials available at minimal procurement costs. Secondly, it reduces the carbon footprint through circular economy implementation. Thirdly, the resultant nanoparticles often retain biologically active trace elements (e.g., Fe and Zn) that enhance nanoparticle functionality.

Recent studies validate these benefits across multiple systems. Mor et al. (2017) achieved 98.9% pure porous SiO_2_ NPs with an average diameter of 10–15 nm using rice husk ash-derived precursor. Similarly, Rangaraj et al. (2017) synthesized amorphous SiO_2_ NPs (diameters of 10–60 nm) from bamboo leaf ash, which maintained > 70% viability in MG- 63 cells at therapeutic concentrations (< 125 mg/L). The integration of waste-to-nanomaterial conversion with green chemistry principles positions this method as a scalable and eco-efficient solution for nanomanufacturing, particularly in applications such as cost-effective adsorbents or drug delivery carriers.

#### Peptide-mediated synthesis

Peptide-mediated biosilicification has redefined precision control in silica nanotechnology, inspired by the molecular machinery of biosilicification observed in marine diatoms. Central to this approach are silica-forming proteins and peptides (SFPs), which typically exhibit high isoelectric points (pI) and are rich in positively charged amino acids, such as lysine (Lys), arginine (Arg), and histidine (His) (Lim & Jo 2025). A representative example is the R5 peptide (SSKKSGSYSGSKGSKRRIL), a bioengineered variant derived from silaffin proteins originally discovered in *Cylindrotheca fusiformis* (Kroger et al. 1999).

The R5 peptide enabled rapid silicic acid polycondensation under physiologically benign conditions (pH 7.0–8.0, room temperature), achieving > 90% silica conversion within 5 min (Senior et al. 2015). The resulting SiO_2_ NPs exhibited superior biocompatibility compared to chemically synthesized counterparts, maintaining 100% viability in mouse fibroblast assays at even 500 μg/mL, whereas conventional nanoparticles induced a 20% decrease in cell viability at concentrations as low as 5 μg/mL (Steven et al. 2014). In addition to the R5 peptide, two other natural SFPs, EctP1 and EctP2, identified from the brown alga *Ectocarpus siliculosus*, exhibited significantly enhanced silica mineralization capacities, achieving 30% to 300% higher silica precipitation across pH gradients compared to the R5 peptide. Notably, EctP1 demonstrated superior performance under acidic conditions (pH 6), where R5 displayed negligible silicification activity (Yeo et al. 2017). Another multifunctional SFP, lysozyme, combines silica-guiding capability with intrinsic antibacterial properties. Its high basicity and hydroxyl group density facilitate silica condensation, while its enzymatic activity enables bacterial cell wall hydrolysis and subsequent cytolysis. These dual-functional attributes make lysozyme a versatile candidate for agricultural applications requiring simultaneous pathogen control and bioactive silica formation (Macchiagodena et al. 2024).

Recent advancements have expanded this biomimetic principle to multifunctional hybrid material fabrication. Elastin-like polypeptides (ELPs) are a class of artificially synthesized polymers. They exhibit unique properties including thermoresponsiveness, self-assembly, and high elasticity (Guo et al. 2023). ELPs have been conjugated with R5 peptides to fabricate hybrid nanocomposites featuring core–shell architectures. These systems exhibited monodisperse ELP micelles (diameters < 100 nm) with precisely controlled silica shell thickness, demonstrating discrete particle morphology and size uniformity (Han et al. 2015). Moreover, genetic fusion of R5 peptides with structural proteins enables programmable hierarchical assembly. For instance, chimeric R5-spider silk proteins self-assembled into β-sheet-rich architectures, guiding the formation of silica-organic composite films and fibers (Wong Po Foo et al*.*, 2006). Further innovation was exemplified by CsgA-R5 amyloid scaffolds, which self-assembled into self-supporting porous structures (1 cm × 1 cm × (412 ± 15) nm, pore size: ~ 10 μm), through in situ mineralization. These composites exhibited excellent mechanical properties, with a threefold increase in Young's modulus after mineralization (from 4.84 ± 0.46 GPa to 14.16 ± 0.58 GPa) (Li et al*.*, 2021).

This interdisciplinary synthesis platform bridges molecular biology and nanotechnology, providing scalable routes for engineering multifunctional silica composites. The resultant materials demonstrate promising applications in catalysis, environmental remediation, and precision drug delivery.

### Comparative analysis and strategic selection of SiO_2_ NP synthesis methodologies

Contemporary SiO_2_ NP synthesis methods exhibit distinct technical trajectories shaped by their foundational mechanisms. ‌Physical and chemical approaches‌ (e.g., sol–gel, flame pyrolysis) dominate industrial-scale production, offering rapid reaction kinetics (< a few days synthesis cycles) and precise stoichiometric control (< 30% size variation) (Wu et al. 2013). However, these methods incur substantial sustainability costs due to energy-intensive conditions and significant carbon emissions. The reliance on cytotoxic surfactants like cetyltrimethylammonium bromide (CTAB) exacerbates environmental risks, with studies showing its toxicity to human osteoblasts and keratinocytes even at a low concentration of 0.2 mM (Carvalho et al. 2022).

Biosynthetic platforms‌ counter these limitations through biologicallly mediated precision. Diatom-derived silaffin peptides achieve ambient-temperature silicification (20–25 °C) with atomic-level replication of pore structures. These systems offer significant environmental advantages, such as agricultural waste valorization (e.g., extracting silica from rice husks at a cost of about $1.33/kg), which simultaneously reduces production costs and environmental burdens (Nandiyanto et al. 2020). The genetic programmability of biomolecular templates further allows modular material engineering. For instance, the R5 peptide can be fused with other proteins (e.g., CsgA and spider silk proteins) via genetic engineering, which is a feature unattainable through traditional synthesis. For cell-based or extractive biosynthesis, process optimization and bioreactor engineering represent critical scalability determinants. Automated continuous-flow bioreactors integrating real-time monitoring systems enable synchronized cultivation of biosynthetic agents (e.g., Saccharomyces cerevisiae engineered for SiO_2_ NP synthesis), nanoparticle production, and downstream applications like nutrient recovery from agricultural runoff (Ali et al. 2024).

Despite the notable advantages of biosynthetic approaches, their agricultural implementation requires careful evaluation of scalability and economic feasibility. For instance, in the case of synthesis using biological cells or extracts, process optimization and bioreactor design represent critical scalability determinants. Automated continuous-flow bioreactors equipped with real-time monitoring systems enable synchronized cultivation of biosynthetic agents, nanoparticle production, and downstream processes (such as wastewater treatment), thus improving production efficiency and reducing operational costs (Ali et al. 2024). In contrast, approaches employing natural biomaterials prioritize feedstock accessibility and pretreatment efficiency. Agricultural residues, such as rice husks, can either undergo localized preprocessing or be transported to centralized facilities for further treatment. Optimization of pretreatment processes, such as drying, alkaline delignification, and mechanical milling, is essential to minimize energy input and overall production cost (Malpani & Goyal 2023). Peptide-mediated synthesis, which is based on the principles of genetic engineering, also holds considerable potential for industrial scalability. For example, through synthetic biology techniques, microbial cell factories can be engineered to express SFPs and cultured in large-scale fermenters, enabling efficient and cost-effective mass production.

The selection of an appropriate synthesis method for SiO_2_ NPs requires a multidimensional evaluation. ‌Agriculture-centric applications‌ favor biosynthesis for enhanced soil compatibility and smart delivery capabilities, while industrial manufacturing retains physical and chemical methods for scalability (> 10,000 ton/year throughput). Hybrid systems that integrate electrochemistry or microwave activation with traditional chemical methods are emerging as promising solutions. These systems prove to be capable of improving synthesis efficiency, slashing reaction times from several hours to dozens of minutes, and enabling precise size (20–500 nm) and morphology control (mesoporous or non-mesoporous) through parameter modulation (Ding & Su 2015, Díaz de Greñu et al*.*, 2020, Snoussi et al. 2018).

## Challenges in the agricultural application of SiO_2_ NPs‌

The integration of SiO_2_ NPs into agricultural systems confronts multidimensional challenges that demand strategic interventions to ensure their sustainable and safe implementation. This section critically analyzes three key barriers and outlines actionable pathways for advancement.

### Environmental safety concerns

The agricultural application of SiO_2_ NPs raises significant environmental concerns that require comprehensive investigation. Although SiO_2_ NPs demonstrate potential for enhancing crop productivity, their environmental fate and ecosystem interactions remain poorly characterized (Wang et al. 2016). Critical knowledge gaps persist in their degradation kinetics and bioaccumulation pathways, which could inadvertently pose risks to non-target organisms.

In soil environments, most engineered nanomaterials can interact with co-existing contaminants, which often lead to antagonism, reducing contaminant concentrations in crops (Zuverza-Mena & White 2018). Depending on the species characteristics and exposure conditions, these co-contaminant interactions may also promote the transfer of organic pollutants from soil to edible plant parts, causing their accumulation in the food chainand subsequent adverse health effects (Wu et al. 2018). SiO_2_ NPs exhibit bidirectional regulatory effects on pollutant mobility, showing potential to reduce transport of certain contaminants while enhancing others. For instance, the addition of 10 mg SiO_2_ NPs per 8 g soil increased the adsorption of rac-metalaxyl in agricultural soil by ~ 120%, while simultaneously weakening its fluidity (Huang et al. 2017). Similarly, SiO_2_ NPs reduced Cd leaching ability by 36.0% and bioavailability by 54.3%, and caused a maximum 61.9% decrease in Cd accumulation in wheat grains (Wang et al. 2020). Contrary to these findings, a recent investigation demonstrated that SiO_2_ NPs enhanced the removal efficiency of persistent organic pollutants including polycyclic aromatic hydrocarbons (PAHs) and total petroleum hydrocarbons (TPHs) through phytoremediation mediated by *Sedum spectabile*, achieving 2.1- and 3.4-fold increases in dissipation rates respectively (Liu et al. 2023*)*. These contradictory outcomes underscore the necessity for risk-centric evaluations to be complemented with context-specific application strategies that account for pollutant physicochemical properties and nanomaterial-bioenvironment interactions.

Bioaccumulation across trophic levels emerges as another critical challenge. Current evidence suggests that nanoparticles may translocate from soil to crops (e.g., translocation factor of Zn nanoparticles from rice roots to shoots up to 1.5) and the aquatic environments, eventually entering the food chain (Maharramov et al. 2019; Peng et al. 2020). These biomagnification dynamics underscore chronic exposure concerns, particularly as recent mechanistic studies reveal that SiO_2_ NPs induce subcellular perturbations including oxidative stress, endoplasmic reticulum (ER) stress, mitochondrial dysfunction, and autophagy impairment upon cellular internalization. Such disturbances disrupt cellular homeostasis through multiple signaling cascades, ultimately leading to programmed cell death. Of particular significance is their capacity to induce DNA damage, a critical hallmark of genotoxicity that exacerbates long-term health risks (Ding et al. 2023).

Ecotoxicological assessments demonstrate concentration-dependent toxicity patterns, with the concentration reported to cause toxic effects spanning six orders of magnitude (ranging from 0.01–1000 mg/L) across species (Andrade et al. 2023; Abdel-Latif et al. 2021; Manzo et al. 2015). Algae, bacteria, and fish embryos exhibited higher tolerance (EC_50_ > 20 mg/L in most studies), attributed to protective cell walls/chorion. In contrast, fish, bivalves, and insects displayed greater susceptibility, with EC_50_ values below 1 mg/L under specific exposure conditions (Book & Backhaus 2022). Comparative toxicity assessments reveal pronounced interspecies differences in SiO_2_ NP responses. Zebrafish embryos and larvae tolerated 200 mg/L SiO_2_ NPs (diameters of 60–200 nm) for 96 h without mortality, whereas *Danio rerio* adults showed significant reproductive dysfunction at 15 μg/L SiO_2_ NPs (35 nm diameter) through 28-day exposure, evidenced by 28.1% fecundity reduction and 20% antioxidant enzyme (SOD/CAT) induction (Fent et al. 2010; Rashidian et al. 2023). Nevertheless, substantial uncertainties persist regarding human bioaccumulation patterns and dose–response relationships, primarily due to the absence of longitudinal human biomonitoring data and population-level health outcome assessments. This knowledge deficit hinders the establishment of species-specific extrapolation models, particularly for nanomaterials demonstrating nonlinear toxicokinetics in aquatic organisms.

Furthermore, surface-functionalized SiO_2_ NPs, designed to improve agricultural efficacy, may introduce additional complexity: exhibit distinct environmental behaviors compared to pristine SiO_2_ NPs, such as dispersion and aggregation in environmental media, uptake, distribution, and toxicity in organisms (Liu & Sayes 2022). These modifications could affect their persistence, bioavailability, and interaction with biological systems, necessitating separate risk assessments and regulatory frameworks.

### Economic viability and scalability barriers

The translation of SiO_2_ NPs from laboratory synthesis to agricultural deployment faces critical economic and scalability barriers rooted in production paradigms. Conventional methods like CVD, sol–gel processes, and mechanical comminution, require energy-intensive conditions (e.g., 800–1200 °C for CVD), platinum catalysts, and costly precursors, resulting in exorbitantly costly agricultural use. Biosynthetic routes leveraging agricultural waste, such as rice husk silica extraction ($1.33–8.13/kg raw material cost), demonstrate theoretical sustainability but face translational bottlenecks (Nandiyanto et al. 2020). The extraction and purification of SiO_2_ NPs synthesized from biological matrices require the identification of inexpensive biological resources and the development of scalable extraction protocols, which are often resource-intensive. Furthermore, downstream processing steps, such as ultracentrifugation and lyophilization, further increase costs, while low silica conversion efficiency and batch-to-batch inconsistency continue to hinder industrial adoption. In addition to these challenges, structural inconsistencies in the derived nanoparticles, evidenced by a polydispersity index (PDI) > 0.2, compromise their performance in precision agricultural applications (Uda et al*.*, 2021).

To make SiO_2_ NPs economically viable for agricultural use, significant advancements are needed in the optimization of biosynthesis processes, automation, and cost reduction strategies. Only through these advancements can SiO_2_ NPs become a commercially feasible and widely adopted solution in agricultural practices.

### Regulatory fragmentation

The regulatory fragmentation surrounding SiO_2_ NPs presents significant challenges to their global agricultural deployment, with divergent risk assessment paradigms and inconsistent governance frameworks exacerbating implementation challenges. Current regulations exhibit marked regional disparities in definitions, risk classification, and testing protocols. For instance, the European Union requires detailed toxicological data submissions under REACH regulations (Hunt et al., 2025), whereas the U.S. EPA's Toxic Substances Control Act (TSCA) lacks standardized nanoscale characterization criteria (Kumari et al. 2023). This regulatory misalignment forces multinational producers into redundant testing cycles, increasing compliance costs for cross-border market entry.

Disparate risk assessments also persist due to unresolved questions about the bio-nano interactions of SiO_2_ NPs, particularly their translocation across biological barriers and environmental persistence (Meng et al. 2018; Xu et al. 2023a). Compounding these issues, inconsistent technical standards for particle characterization, emission limits, and environmental monitoring impede cross-border regulatory alignment (López-Serrano et al*.*, 2014).

Efforts to establish global governance frameworks emphasize three synergistic pathways. First, international coordination mechanisms are emerging through initiatives like the OECD's standardized toxicity testing protocols and UNEP's Global Chemicals Outlook, which aim to unify chemical inventories and data-sharing platforms (Carnesecchi et al. 2023). Second, advancing foundational research on the structure–property correlations of SiO_2_ NPs is essential, as it provides critical insights for evidence-based policymaking (Tkachenko et al. 2020). Finally, public–private partnerships are driving technological innovations, such as green synthesis methods for SiO_2_ NPs and industry-led environmental standards in construction materials, which help bridge regulatory gaps through voluntary compliance (Karande et al*.*, 2021, Lee et al. 2010).

These strategies collectively address the dual imperatives of enabling the rapid advancement of nanotechnology while mitigating unintended health and ecological impacts through science-driven, adaptive governance.

## Conclusions and future perspectives

In conclusion, SiO_2_ NPs demonstrate multifunctional potential in agricultural innovation through their dual capacity to enhance crop stress resilience and enable precision agrochemical delivery. Their biosynthetic production pathways offer environmentally sustainable alternatives to conventional synthesis methods, though critical challenges persist in environmental safety assessment, cost-effective scale-up, and global regulatory alignment. The current fragmented policy landscape continues to hinder standardized commercialization pathways across jurisdictions.

Future progress necessitates interdisciplinary synergies to simultaneously address environmental, economic, and regulatory challenges. A deeper understanding of their interaction mechanisms with plants, soil microbes, and environmental systems, especially at the molecular level, is essential. It is also important to develop predictive frameworks for environmental impact assessment, which will advance our knowledge of SiO_2_ NPs ecotoxicity and biogeochemical cycling. To achieve cost-effective scalability and improved agronomic performance, innovative synthesis and application technologies such as hybrid synthesis methods and smart agricultural uses should be prioritized. Moreover, fostering international cooperation in data sharing, resources integration, and the establishment of unified regulations is imperative. Building global consensus on nano-agriculture governance will play a pivotal role in ensuring the responsible and sustainable deployment of silica nanoparticles in agricultural systems. By addressing these challenges, SiO_2_ NPs technologies may ultimately catalyze a transformative shift toward sustainable and precision-driven agricultural systems, balancing productivity demands with ecological stewardship.

## Data Availability

No datasets were generated or analyzed during the current study.
